# Unity among the diverse RNA-guided CRISPR-Cas interference mechanisms

**DOI:** 10.1016/j.jbc.2024.107295

**Published:** 2024-04-18

**Authors:** Chhandosee Ganguly, Saadi Rostami, Kole Long, Swarmistha Devi Aribam, Rakhi Rajan

**Affiliations:** Department of Chemistry and Biochemistry, Price Family Foundation Institute of Structural Biology, Stephenson Life Sciences Research Center, University of Oklahoma, Norman, Oklahoma, USA

**Keywords:** CRISPR-cas, crRNA, RNA binding protein, Cascade, Cas3, Cas10, CasDinG, Cas9, Cas12, Cas13

## Abstract

CRISPR-Cas (clustered regularly interspaced short palindromic repeats-CRISPR-associated) systems are adaptive immune systems that protect bacteria and archaea from invading mobile genetic elements (MGEs). The Cas protein-CRISPR RNA (crRNA) complex uses complementarity of the crRNA “guide” region to specifically recognize the invader genome. CRISPR effectors that perform targeted destruction of the foreign genome have emerged independently as multi-subunit protein complexes (Class 1 systems) and as single multi-domain proteins (Class 2). These different CRISPR-Cas systems can cleave RNA, DNA, and protein in an RNA-guided manner to eliminate the invader, and in some cases, they initiate programmed cell death/dormancy. The versatile mechanisms of the different CRISPR-Cas systems to target and destroy nucleic acids have been adapted to develop various programmable-RNA-guided tools and have revolutionized the development of fast, accurate, and accessible genomic applications. In this review, we present the structure and interference mechanisms of different CRISPR-Cas systems and an analysis of their unified features. The three types of Class 1 systems (I, III, and IV) have a conserved right-handed helical filamentous structure that provides a backbone for sequence-specific targeting while using unique proteins with distinct mechanisms to destroy the invader. Similarly, all three Class 2 types (II, V, and VI) have a bilobed architecture that binds the RNA-DNA/RNA hybrid and uses different nuclease domains to cleave invading MGEs. Additionally, we highlight the mechanistic similarities of CRISPR-Cas enzymes with other RNA-cleaving enzymes and briefly present the evolutionary routes of the different CRISPR-Cas systems.

CRISPR-Cas systems are adaptive immune systems that protect bacteria and archaea from invading mobile genetic elements (MGEs) such as viruses and plasmids (1, 2, 3, 4, 5, 6). The presence of palindromic repeats associated with CRISPR was fortuitously identified in *Escherichia coli* in 1987 while studying the function of the *iap* gene (7). A few years later, similar palindromic repeats were observed in archaeal systems (8) and identified as a new family of repeats in prokaryotes (9). In 2002, these repeat sequences were named clustered regularly interspaced short palindromic repeats (CRISPR) and were followed by the identification of the CRISPR-associated (*cas*) genes flanking the CRISPR locus (10).

Several studies have established the unified features of the CRISPR-Cas interference mechanisms. These attributes included the establishment of a CRISPR array as a collection of palindromic “repeats” that are interspaced by short DNA sequences called “spacers,” which serve as molecular memories of previous encounters with foreign genetic materials (1, 2, 11). While CRISPR-Cas systems were initially predicted to play a role in DNA repair (12), the first experimental evidence of their role in protecting bacteria from invading phages was demonstrated in *Streptococcus thermophilus* in 2007 (5). In addition, the Cas proteins were shown to bind to the CRISPR RNA (crRNA), which is transcribed and processed from the CRISPR array, and to enable targeted destruction of the invading MGEs (5, 6, 13).

CRISPR-Cas systems are classified by their protein and RNA components, the targets they destroy, and the type of immune protection they offer to their host cell (14, 15). These diverse systems are broadly divided into two major classes depending on the presence of multiple (Class 1) or single (Class 2) Cas protein bound to the crRNA in the surveillance complex (15, 16). The classes are further divided into six distinct types based on a signature protein that is often the nuclease that inactivates the invading genetic element (14). Types I, III, and IV of Class 1 have the signature proteins Cas3 (17, 18), Cas10 (19, 20), and CasDinG/CysH-like/Cas10-like (21), respectively. Types II, V, and VI of Class 2 are characterized by the presence of Cas9 (5, 22), Cas12 (23), and Cas13 (24), respectively. Each type is further divided into several subtypes based on the CRISPR-Cas locus composition that contributes to unique mechanisms and functional variations (14, 15, 16) (Table 1, Figs. 1 and 2). The diversity of CRISPR-Cas systems is constantly growing due to the addition of new and rare CRISPR types that are being identified from genome and metagenomic data analyses (25, 26).

**Table 1:** Classification and features of CRISPR-Cas subtypes

Class/Type	Subtype	Signature protein/effector	gRNA & processing enzyme	Primary target	Other activities	Nuclease Domain(s)	Ref
Class 1, type I	A-G	Cas3	crRNA, Cas6 (only I-C uses Cas5d)	dsDNA	Unknown (except for I-B1 and I-B2, which exhibit DNA transposition)	Cas3: HD domain	(97, 105, 107, 109, 110, 111, 115, 121, 337)
	F3 (CAST)	Lacks nuclease	crRNA, Cas6f	dsDNA	Transposition of DNA	Cas3 is absent, transposition nuclease is present	(34)
Class1, type III	A-D and F	Cas7, Cas10	crRNA, Cas6 (III-A and B), unknown for other subtypes	Cas7: ssRNA, Cas10: non-specific ssDNA	Non-specific RNA cleavage by Csm6/Csx1	Cas10: HD domain Cas7: a catalytic loop	(339, 360)
	E	Cas7-11 fusion protein, TPR-CHAT	crRNA, Possibly host nucleases	Cas7 domain: ssRNA cleavage TPR-CHAT: protease	Unknown	Cas7 domain: catalytic loop; TPR-CHAT for protease activity	(28)
Class1, type IV.	A	CasDinG (HNH insertion for some systems)	crRNA, Cas6	dsDNA (plasmid)	Unknown	CasDinG-HNH domain insertion for some species	(21, 26, 217)
	B	CysH-like	heterogeneous RNA, Unknown	Unknown	Unknown	Unknown	
	C	Cas10-like	Unknown	Unknown	Unknown	Unknown	
	D	RecD	crRNA, Cas6	Unknown	Unknown	Unknown	
	E	CasDinG	crRNA, Cas6	Unknown	Unknown	Unknown	
Class2, type II	A-D	Cas9	crRNA+ tracrRNA, Host RNase III+ Cas9	dsDNA	None	HNH and RuvC	(15, 25)
Class2, type V	A	Cas12a	crRNA, Cas12a by WED domain	dsDNA and ssDNA	ssDNA *trans-* cleavage	RuvC	(23, 290)
	B	Cas12b	crRNA+ tracrRNA, Unknown	dsDNA and ssDNA	ssDNA *trans-*cleavage	RuvC	(335, 361)
	C	Cas12c	crRNA+ scout RNA, Cas12c by RuvC	Varying reports of transcription repression and *cis*-DNA cleavage	Varying reports of ssDNA *trans*-cleavage	RuvC	(142, 155, 241, 292, 362)
	D	Cas12d (CasY)	crRNA+ scoutRNA, Unknown	dsDNA and ssDNA	ssDNA *trans-*cleavage	RuvC	(142)
	E	Cas12e (CasX)	crRNA+ tracrRNA, Unknown	dsDNA	ssDNA *trans-*cleavage	RuvC	(363)
	F	Cas12f	crRNA+ tracrRNA, unknown	dsDNA and ssDNA	ssDNA *trans-*cleavage	RuvC	(364)
	G	Cas12g	crRNA+ tracrRNA, Unknown	ssRNA	ssRNA and ssDNA *trans-*cleavage	RuvC	(292, 365)
	H	Cas12h	crRNA, Cas12h domain unknown	dsDNA and ssDNA	ssDNA *trans-*cleavage	RuvC	(292)
	I	Cas12i	crRNA, Cas12i by WED domain	dsDNA and ssDNA	ssDNA *trans-*cleavage	RuvC	(292)
	J	Cas12j (Cas Φ)	crRNA, Cas12j by RuvC domain	dsDNA and ssDNA	ssDNA *trans-*cleavage	RuvC	(39)
	K	Cas12k	crRNA+ tracrRNA, Unknown	dsDNA	Functions as CAST	Inactive	(35)
	L	Cas12l (Cas π)	crRNA+tracrRNA, Unknown	dsDNA and ssDNA	ssDNA *trans-*cleavage	RuvC	(152)
	M	Cas12m	crRNA, Cas12m by WED domain	dsDNA	Function as transcription repressor	RuvC (doesn’t cleave target)	(153)
Class2, type VI	A-D	Cas13	crRNA, Cas13	ssRNA	non-specific ssRNA	HEPN	(258, 259)

**Figure 1:**
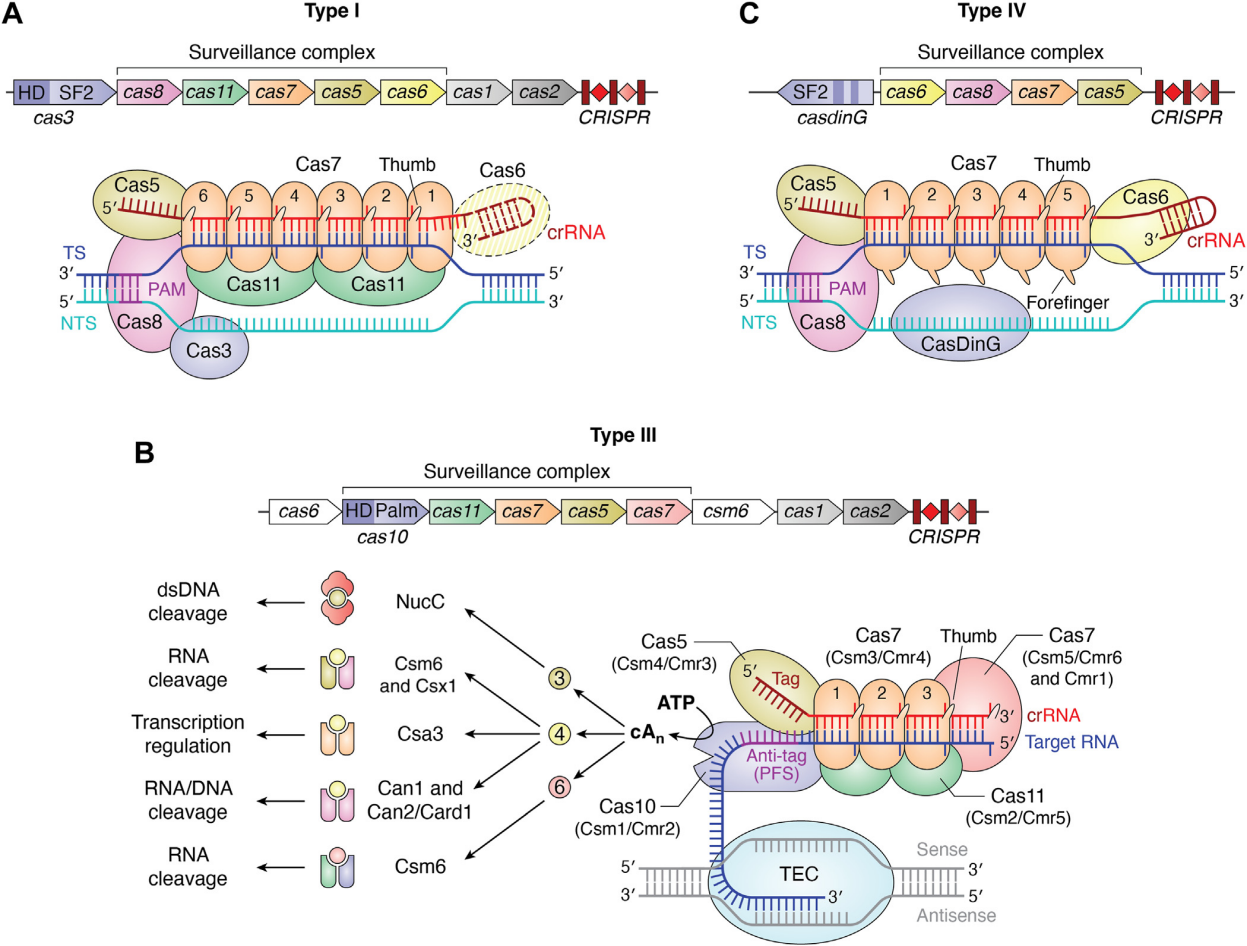
**Schematic representation of a typical CRISPR-Cas locus (top) and the overall interference complex organization (*bottom*) for each type belonging to the Class 1 system.** The CRISPR array with repeats and spacers is shown in crimson and red. The target strand (TS) of the nucleic acid is in *blue*, the non-target strand (NTS) is in *cyan*, crRNA is in *red*/crimson and the recognition motif (PAM/PFS) is in *dark purple* for all the systems. *A*, for the type I system (subtype I-E is shown here), Cas6 bound to the 3′ stem-loop of crRNA is represented in *dotted outline* since it is not part of the helical filament for certain subtypes (I-A, I-B, and I-D). Cas3 nuclease is a part of the surveillance complex before DNA recognition in some subtypes whereas in others it is recruited after R-loop formation. *B*, the type III systems (subtype III-A complex is shown here) are broadly categorized into III-A (Csm) and III-B (Cmr) subtypes. In the example depicted, a type III surveillance complex binds to the nascent RNA (target) produced by the RNA polymerase, near the transcription elongation complex (TEC). After binding to a non-self RNA target, Cas10 present in the complex becomes activated for ssDNA cleavage by the HD domain and cOA production by the Palm domains. The cA_n_-dependent activation of ancillary proteins initiates non-specific cleavage. *C*, the organization of the type IV-A surveillance complex on a mature crRNA recruit CasDinG for ATP-dependent DNA unwinding. In all the types, the periodically flipped out crRNA nt can be seen near the thumb domain.

**Figure 2:**
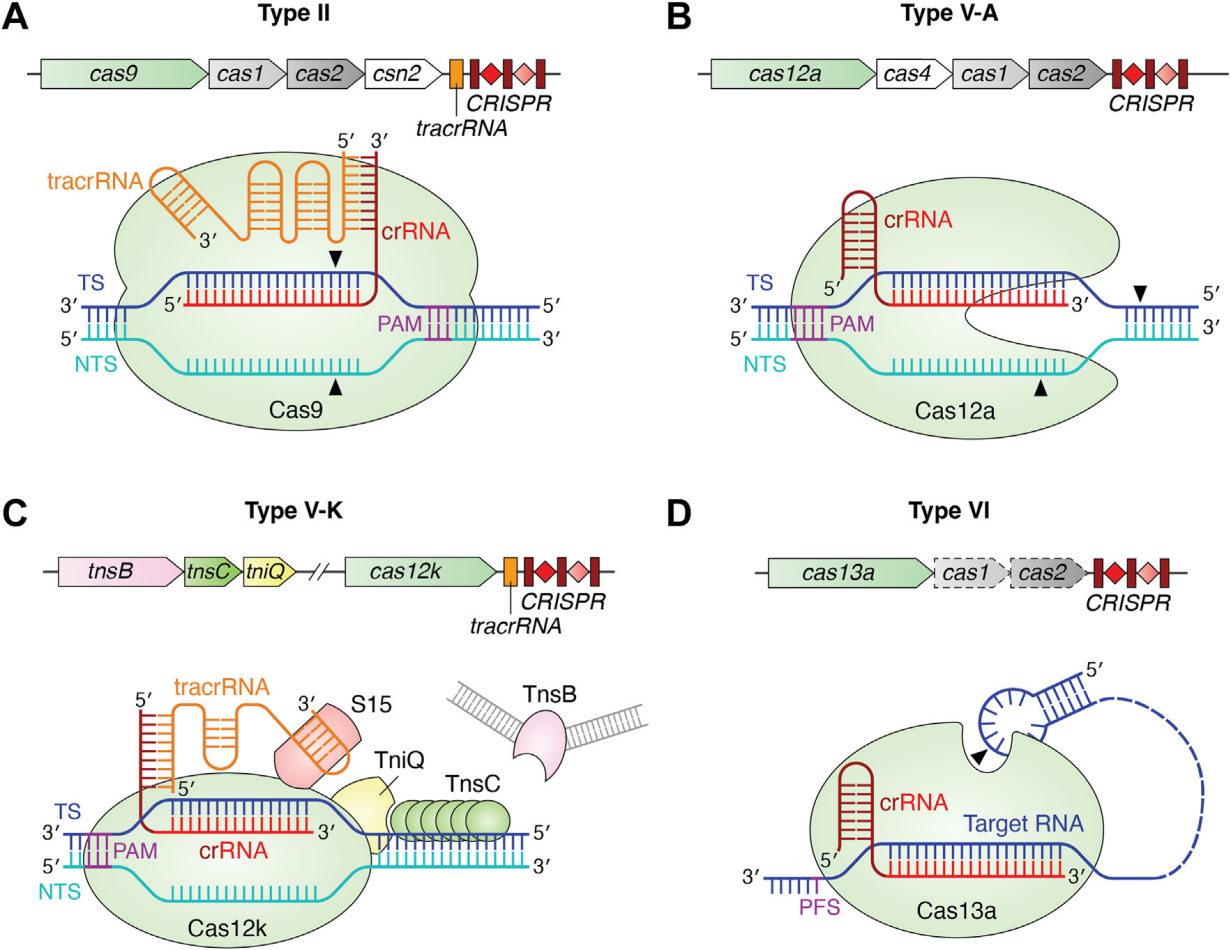
**Schematic representation of a typical CRISPR-Cas locus (top) and the organization of the interference complex (*bottom*) for each type belonging to the Class 2 system.** The color scheme is as follows: the effector protein in *mint green*, crRNA in *red*/crimson, tracrRNA in *orange*, TS in *blue*, NTS DNA in *cyan*, and the recognition motif (PAM/PFS) in *dark purple*. *Dashed outlines* show dispensable genes. *Black triangles* represent cleavage sites. *A*, the bilobed type-II Cas9 nuclease (subtype II-A is shown here) assembles with the crRNA and the tracrRNA, followed by targeting and cleavage of dsDNA. *B*, the type-V Cas12 nuclease (subtype V-A is shown here) binds to the crRNA and cleaves NTS and TS DNA sequentially. *C*, the ShCAST DNA transposition complex utilizes a catalytically inactive Cas12k for crRNA-tracrRNA dependent and PAM-specific DNA targeting and transposition. Other components such as TnsB, TniQ, and the ribosomal protein S15 are essential for effective transposition. *D*, the type VI Cas13 nuclease (subtype VI-A is shown here) bound to the crRNA cleaves ssRNA. The *dashed region* of the target RNA represents the variations in its length and structure.

Advances in the previous 2 decades have revealed wide-ranging mechanisms of RNA-guided immune protection offered by CRISPR-Cas systems and the counter mechanisms that were developed by viruses. Some examples include sequence-specific cleavage of invading DNA and RNA genomes (5, 13, 19, 27) and proteins (28, 29), destruction of invader genome *versus* altruistic destruction of bacterial cells (30, 31, 32), co-option of CRISPR systems and transposons for site-specific DNA transposition (33, 34, 35, 36, 37), hijacking of CRISPR systems by huge phages to destroy smaller phages (38, 39, 40), and the use of anti-CRISPR proteins and small non-coding RNA anti-CRISPRs (41, 42, 43, 44) by the viruses to evade CRISPR-Cas immune protection. The multitude of functions is made possible by adapting only slight changes in the basic design principle of RNA-guided nucleic acid-targeting by Cas proteins, which modulate the enzymatic activity of specific Cas protein(s) to bring about the physiological outcome. The ease of targeting by CRISPR-Cas systems has made it a powerful tool in genome engineering and molecular diagnostics (45, 46, 47, 48). To expand the repertoire of applications in molecular medicine, CRISPR-Cas systems are being adapted for use in gene therapy, either alone or in combination with other molecular systems to create tools such as prime and base editors (16, 49, 50, 51, 52, 53, 54, 55).

Here, we aim to provide an overview of the diverse CRISPR-Cas systems, highlighting the similarities in their subunit arrangement, structure, and interference mechanisms. Interestingly, in some cases, the same basic mechanism is further fine-tuned to enable alternate functions such as bacterial pathogenicity and cell signaling, details of which can be found in recent reviews (15, 50, 56, 57, 58, 59). We also compare the catalytic mechanisms of the interference nucleases and discuss the evolutionary relationships between the different CRISPR types.

## Stages of CRISPR-Cas defense

The overall cycle of CRISPR-Cas systems in bacteria and archaea functions in three major stages. In the *first* stage, adaptation, a fragment from the invading nucleic acid, called a spacer, is excised and integrated sequence specifically into the CRISPR array by Cas1-Cas2 proteins (1, 2, 11, 60, 61, 62). A suite of Cas and other cellular proteins (*e.g.*, RecBCD, AddAB, SbcCD, Integrated Host Factor) are essential for spacer excision from the invader genome and for sequence-specific insertion into the CRISPR locus. The mechanism of this process varies for the different types of CRISPR-Cas systems (63, 64, 65, 66, 67). The *second* stage is crRNA processing, where the pre-crRNA transcripts transcribed from the CRISPR-locus are processed into individual crRNAs by designated Cas enzymes or cellular nucleases (6, 68, 69, 70, 71). The crRNA then complexes with one or more Cas protein(s) to form a surveillance (effector) complex that surveys the cell and monitors the entry of foreign nucleic acids (19, 72). In the *third* stage, the surveillance complex uses a “guide” sequence within the crRNA to locate a complementary DNA and/or RNA target (termed a protospacer), forming an interference complex. The interference complex leads to the formation of an R-loop [RNA-DNA hybrid where one strand (target strand, TS) of the DNA base pairs with the guide region of the crRNA and the other one (non-target strand, NTS) exists as a single strand] or an RNA-RNA hybrid based on the CRISPR type (Figs. 1 and 2) (19, 73, 74).

Specific sequence motifs that are present in the target DNA flanking the protospacer (*e.g.*, protospacer adjacent motif, PAM) (21, 23, 75, 76, 77, 78, 79) or RNA (protospacer flanking sequence, PFS) (30, 80, 81, 82) enable the discrimination of self-*versus* non-self-genomes. Within the guide region of the crRNA, a short stretch of sequence called the “seed” is critical in initiating the hybridization of the crRNA with the target. A strict sequence complementarity between the crRNA-guide and the protospacer is essential within the seed region to activate the system for target cleavage (83). However, the length of this seed and the extent of seed exposure in the surveillance complex to facilitate target search vary between the different CRISPR types (30, 72, 77, 83, 84, 85). The formation of an interference complex with a fully paired guide and target triggers cleavage of the invading element by specific nuclease(s). Based on the CRISPR type, the nuclease activity can degrade the invading DNA and/or RNA transcripts to inactivate the infection (5, 6, 23, 30, 77, 86, 87). In certain systems, signaling mechanisms are activated resulting in the synthesis of small cyclic oligonucleotides, which in turn activate ancillary nucleases and trigger dormancy or death of the host cell (88).

The abovementioned stages of CRISPR-Cas defense mechanisms are a common theme, but variations are found in the case of “primed adaptation,” where a faulty interference process can enable the uptake of new spacers from an invader (89, 90). Primed adaptation commonly occurs when the interference complex assembles over a mutated protospacer, attenuating the cleavage of the invader. However, the stalled complex promotes the uptake of new spacers into the CRISPR array more efficiently than a naïve adaptation process (91, 92, 93).

## Formation of surveillance (effector) complexes

The general mechanism of surveillance complex formation involves the assembly of Cas protein(s) and the mature crRNA. The biogenesis of crRNA occurs either before (in most Class 1 systems) or concurrently (Class 2) with the formation of the surveillance complex based on the CRISPR type (71). The pre-crRNA transcribed from the promoter region of the CRISPR locus is processed into mature crRNA consisting of a spacer sequence flanked by partial repeats (6, 68, 94). In this section, we discuss the details of the organization of the surveillance complexes of Class 1 and Class 2 systems.

### Helical filamentous assembly of Class 1 systems

In most Class 1 systems, Cas6 is responsible for processing pre-crRNA to generate a mature crRNA that is 60 to 70 nucleotides (nt) in length with a 3′-stem-loop and a 5′-handle (4, 6, 68, 95, 96). The Class 1 complexes organize as a right-handed helical filamentous structure over the mature crRNA (97, 98, 99) (Fig. 1). All three types of Class 1 systems contain a helical assembly consisting of a major filament composed of Cas5 and Cas7 proteins, which interacts with a minor filament composed of several Cas11 subunits and a single large subunit; Cas8 in types I/IV or Cas10 in type III (73, 100, 101, 102). Cas5 and Cas7 are RNA-binding repeat-associated mysterious proteins [RAMPs; they contain RNA Recognition Motifs (RRM) with glycine-rich loops] (103, 104). The structure of Cas7 resembles a right hand consisting of fingers, a palm, and a thumb domain. The Palm domain contains an RRM that binds non-sequence specifically to the guide region of the crRNA (105, 106). A distinguishing feature of Class 1 complexes is the flipping out of every sixth nt of the guide region of the crRNA. The thumb and the finger domains stabilize the flipped-out nucleotide (106) (Fig. 1). Structural studies have shown that the Cas7-like proteins in the different Class 1 subtypes bind to crRNA with different torsional angles at the sixth nt, resulting in kinks and leading to large variations in their helical pitches (106, 107, 108). A single copy of Cas5 binds to the 5′-end of the crRNA.

Post-crRNA maturation, Cas6 dissociates from the surveillance complex of most subtypes (I-A, I-B, I-C, I-D, type III-A, and III-B) (101, 109, 110, 111, 112, 113), but in some systems (subtypes I-E, I-F, I-G, and type IV-A) (98, 107, 114, 115) it can stay associated at the 3′-stem-loop of the crRNA (Fig. 1). In certain types (*e.g.*, most type Is, III-A, III-B, and IV-A systems), Cas6 is present within the CRISPR-Cas locus while in others the crRNA processing ability of a single Cas6 is shared with coexisting CRISPR systems that lack Cas6 in the same host [*e.g.*, two type III-Bs sharing crRNA from a type I-A system in *Sulfolobus islandicus* (116), and types III-B and I-G sharing crRNA processed by I-A of *Pyrococcus furiosus* (117)] or is substituted by another protein (*e.g.*, in *Bacillus halodurans* and *Mannheimia succiniproducens* harboring the type I-C system, Cas5d was observed to process pre-crRNA) (118, 119).

#### Type I

The seahorse-shaped type I-E helical filament was the first to be structurally resolved, and it was originally referred to as the CRISPR-associated complex for antiviral defense (Cascade) (6, 73, 105, 114, 120). Type I CRISPR-Cas system consists of several subtypes (I-A to I-G) with variations in the surveillance complex composition and appearance as well as the presence/absence of *cas6* in the locus (14, 15). All of them consist of a major and a minor filament. The number of Cas7 subunits in the major filament varies between six to eight based on the CRISPR subtype and the length of the guide region of the crRNA (32–50 nt) (97, 107, 109, 110, 111, 114, 115, 121). In the subtypes I-A (122, 123), I-B (110), and I-D (111), wherein Cas6 dissociates from the surveillance complex, the stem-loop at the 3′-end of the crRNA is trimmed. The minor filament consists of two to five Cas11 subunits and a Cas8 subunit (variation among subtypes is shown in Table 2, Fig. 1*A*). It has been proposed that the Cas5-Cas7 heterodimer recognizes and binds to the 5′-handle of the crRNA to trigger nucleation and subsequent polymerization of Cas7 proteins (120), which in turn forms the right-handed helical backbone of the surveillance complex. The 3′-stem-loop of the crRNA on the other end plays a role in terminating Cas7 polymerization. The Cas8 subunit, which physically associates with the Cas5 and one of the Cas7 subunits (Fig. 1*A*) is responsible for PAM recognition during target DNA search (124).

**Table 2:** Diversity of type I CRISPR-Cas systems

Type I subtype	Cas7	Cas11	Unique features	Ref
A	7	5	• *cas3*′ (helicase) and *cas3*″ (HD-domain) are two separate genes • Association of Cas3*′* and Cas3″ with the Cas8g subunit is necessary for DNA recognition (Cas3″ is part of helical assembly in an autoinhibited stage) • Complete R-loop formation allosterically activates Cas3*′* and Cas3″ for processive DNA degradation	(109)
B	6-8	2	• Single gene encodes for Cas3 subunit • Some type I-B (I-B1 and I-B2) families encode Tn-7-like CRISPR-associated transposon (CAST) genes: *tniQ*/*tnsD* homologs	(110, 366)
C	7	2	• Single gene encodes for Cas3 subunit • Uses Cas5 for crRNA processing • *cas11* is encrypted towards the 3′-end of the *cas8* gene	(121)
D	7	2	• Cas10d-Cas3″ and Cas3′ are encoded by two separate genes. • Cas10d-Cas3″ fusion protein replaces Cas8 • Cas10d domain recognizes the PAM and undergoes conformational changes activating Cas3″ domain • Activated Cas3″ domain recruits Cas3′ and both are needed for processive DNA degradation	(111, 367)
E	6	2	• Single gene encodes for Cas3 subunit • Cas8 large subunit and Cas11 forms the minor filament • Cas3 is *trans-*recruited after complete R-loop formation	(105)
F1	6	0	• The *cas2* and *cas3* genes are fused and produces a single polypeptide • Minor filament is absent due to the lack of Cas11 subunits • Cas8f is present and is involved in PAM recognition • Upon binding to target DNA, the surveillance complex undergoes ∼20 Å elongation to accommodate the RNA:DNA hybrid.	(107, 339)
F2 (v)	6	0	• Cas5 and Cas7 substitute for the missing Cas8 and Cas11 subunits • Cas5 facilitates PAM recognition • Finger domain in Cas7 is extremely reduced, instead there are two extended loops that assembles into a unique helical filament termed wrist helix • Cas5 also has a wrist that connects to the Cas7 wrist helix	(97)
F3	6	0	• Lacks Cas3 helicase-nuclease • In addition to the regular type I *cas* genes, type I-F3 encodes *tnsQ.* • Transposase genes (*tnsA, tnsB, tnsC*, and *tnsD*) are located near the type I-F3 operon separated by a cargo region. • Helical filament co-complexes with the transposition protein, TniQ	(368)
G	7	0	• A single gene encodes for Cas3 • Association of Cas3 with Cas8g is important for DNA recognition • The large Cas8g subunit spans the entire belly region	(115)

The Cas3 protein, which possesses a histidine-aspartate (HD) nuclease domain and a Superfamily 2 (SF2) helicase domain, processively degrades the invader DNA (125, 126, 127, 128). Two forms of associations of Cas3 have been observed for type I surveillance complexes; one that includes *trans-*recruitment of Cas3 after locating the target DNA for cleavage (*e.g.*, subtypes I-B, I-C, I-E, and I-F) and the other where an autoinhibited Cas3 being part of the surveillance complex that locates the target DNA (*e.g.*, subtypes I-A, I-D, and I-G) (discussed in Catalytic mechanisms related to CRISPR-Cas interference).

#### Type III

The type III CRISPR-Cas system consists of several subtypes (III-A to III-F) with variations in the surveillance complex composition and appearance as well as the presence/absence of the *cas6* gene and CRISPR array in the locus. The two distinct subtypes of type III are based on the features of Cas11: the Csm complex (Cas subtype M, comprising subtypes A, D, E, and F, with Csm2 featuring as Cas11) and the Cmr complex (Cas module RAMP, composed of subtypes B and C, with Cmr5 featuring as Cas11) (14, 129). The ∼60 to 71 nt-long crRNA produced by Cas6 is further trimmed at the 3′-end by host nucleases generating mature crRNA ranging in length between 31 to 46 nt and with varying numbers of bound Cas7 subunits based on the length of the crRNA (13, 71, 95, 130). Examples of host nucleases involved in trimming the 3′-end are the degradosome-associated nucleases such as PNPase and RNaseJ2 in type III-A (131). Studies have shown that the type III-B system that does not contain a CRISPR array uses the crRNA produced from type I-F’s CRISPR array to provide synergistic immune protection (117). The helical filament of type III systems is slightly extended compared to that of type I and has a worm-like appearance (108, 132, 133). The type III major filament consists of one non-catalytic Cas7 (pink in Fig. 1*B*) and two to four catalytic Cas7-like proteins (light orange in Fig. 1*B*) interacting with the guide region of the crRNA, and one Cas5 protein capping the 5′-repeat tag of the crRNA. The minor filament consists of one Cas10 and two to three Cas11 subunits (101, 108, 129) (Fig. 1*B*). The signature protein Cas10 is composed of two Palm domains and an HD nuclease domain. The filaments are tethered together by the interaction between Cas7-like and Cas11 proteins at the head region and Cas5 and Cas10 proteins at the tail region of the surveillance complex (108, 132, 133) (Fig. 1*B*).

#### Type IV

The type IV systems have very distinct organizational features between the subtypes (IV-A to IV-E) (21) based on the composition of the major and minor filaments and the signature protein (*e.g.*, CasDinG, CysH-like, Cas10-like, and RecD proteins in subtypes IV-A and E, IV-B, IV-C, and IV-D respectively) (15, 134). Recently, more subtypes with unique features have been identified for the type IV systems (26, 135). The subtypes also differ in the presence/absence of the CRISPR array and Cas6 (15, 21, 135). Studies have shown that while the type IV-A system’s helical filament assembles on a 61 nt-long cognate crRNA (98), the type IV-B filament assembles on a non-crRNA (136). Most type IV helical filaments consist of Cas8-like, Cas7, and Cas5 proteins (Fig. 1*C*). In addition to these proteins, Cas11 is present in types IV-B and IV-C (14, 21, 136, 137). The seahorse-like architecture of the type IV-A system (98) resembles the type I-F surveillance complex (107). However, the recently resolved sea cucumber-like helical filament of the type IV-B system is similar to that of the type III system (136). These differences in helical filament structures and the signature protein identity exemplify the striking diversity among the type IV subtypes.

### Bilobed organization of Class 2 systems

The Class 2 CRISPR systems comprise a single multi-domain protein that forms the surveillance complex after binding to a guide RNA (gRNA) (15, 104). (Fig. 2). The surveillance complex has a compact bilobed architecture, which is a conserved feature of all Class 2 effector proteins (except type V-M, which possesses a unique bracelet-like architecture) (138, 139, 140). Unlike Class 1, Class 2 systems do not have Cas6 to process the pre-crRNA. Instead, they use distinct mechanisms for crRNA processing ranging from the direct RNase activity of an effector Cas nuclease (*e.g.*, Cas12a in type V-A) (23) to the use of host-encoded RNases (*e.g.*, RNase III in type II) (69) to process its crRNA. In certain Class 2 systems, other RNAs such as the *trans-*activating CRISPR RNA (tracrRNA in type II and type V-B; Table 1) (141) or short complementarity untranslated RNA (scoutRNA in type V-D) (142) hybridize with the pre-crRNA to mediate crRNA processing either by the Cas effector or by the host nucleases.

The distinctive feature of type II and V signature proteins, Cas9 and Cas12, respectively, is their characteristic bilobed architecture in which the lobes are connected by a bridge helix (138, 140). The recognition (REC) lobe binds to the cognate gRNA and the nuclease (NUC) lobe houses the sequence-specific PAM interacting (PI) domain and the endonuclease domain(s): HNH (a motif characterized by Histidine and Asparagine residues) and/or RuvC (Holliday junction resolvase like domain with RNase H-fold) (138, 140). Both types II and V cleave DNA targets (15). Cas13, the signature protein of type VI, does not share significant sequence similarities with Cas9 and Cas12 but displays a compact bilobed architecture (139, 143). Cas13 possesses higher eukaryotes and prokaryotes nucleotide-binding (HEPN) domains to cleave single-stranded RNA targets (15).

#### Type II

The type II CRISPR-Cas system is divided into four subtypes (II-A to II-D) based on their distinct operon organization and features of the effector nuclease (15, 25, 144). The type II-A Cas9 protein from *Streptococcus pyogenes* (Sp) is the most extensively studied Class 2 system. Type II systems require tracrRNA, which base pairs with the repeat region of crRNA through its anti-repeat region for all the three stages of the CRISPR-defense mechanism (Fig. 2*A*) (77, 145). Cas9 binds to pre-tracrRNA and pre-crRNA that recruits RNase III, resulting in the concurrent maturation of both tracrRNA and crRNA (69). The surveillance complex is composed of a Cas9-crRNA-tracrRNA complex, which adopts the bilobed architecture (138, 146, 147). The 20 nt-long spacer-derived sequence of the crRNA is the guide that enables Cas9 to locate a target DNA based on complementarity with the guide region (77, 138). The structure of Cas9 bound to a single gRNA (sgRNA, created by fusing crRNA and tracrRNA with a tetraloop) induces significant domain rearrangements to promote a Cas9 conformation that is ready for target DNA search (148). The major conformational change occurs in the REC-III domain that moves ∼65 Å towards the HNH domain, forming a central channel of ∼25 Å width between the two lobes (148, 149). Structural comparisons also showed that the bridge helix connecting the two lobes of Cas9 undergoes a loop-to-helical transition upon sgRNA binding (138, 146, 149), which is important for DNA cleavage (150, 151).

#### Type V

The type V CRISPR-Cas systems (subtypes V-A to V-M) are the most diverse among the currently classified Class 2 systems (Table 1) (15, 16, 152, 153). The unifying theme among these diverse type V CRISPR systems is the presence of a single RuvC-like endonuclease domain within the bilobed architecture of the Cas effector protein (15). The surveillance complex of the type V subtypes is composed of Cas12 and gRNA (crRNA or crRNA+ tracrRNA/scoutRNA) (Fig. 2, *B* and *C*). Pre-crRNA processing occurs in three different ways depending on the components. In type V-A system, Cas12a self-processes at the 5′-end of its crRNA (154). However, some others, such as type V-C and V-D, use Cas12’s 3′-self-processing activity guided by scoutRNA or rely on host RNase III to process the crRNA-tracrRNA hybrid bound to Cas12, as seen in type V-B and V-E (142, 155) (Table 1). The apo-protein exists in an equilibrium between open and closed states. Upon binding to pre-crRNA, the binary complex transitions into the conventional closed and compact bilobed conformation with a positively charged channel in the interface for RNA/DNA binding (156).

#### Type VI

Type VI systems comprise four subtypes (VI-A to VI-D) based on their locus organization. The characteristic feature is the presence of two HEPN domains among all the subtypes, which are very divergent in sequence (104). The effector protein of type VI possesses two distinct RNA cleavage sites, one for processing the pre-crRNA and another for cleaving target RNA (139, 157, 158). Cas13a, the initial Cas endonuclease identified from type VI (30), was observed to self-process its crRNA. During self-processing, Cas13a cleaves the 5′-end of the pre-crRNA within the repeat region and remains bound to the mature crRNA to form the surveillance complex (Fig. 2*D*) (139, 157, 158). Similar to other Class 2 systems, conformational changes occur in Cas13a when it binds to crRNA, forming a central channel capable of accommodating the crRNA: target RNA duplex (139, 157).

## Inactivation of invaders by the interference complex

The surveillance complex surveys the cell for the presence of an invading MGE. The invading genome is located based on the presence of a PAM or a PFS in the foreign nucleic acid, leading to the formation of an R-loop or an RNA-RNA hybrid based on the CRISPR type (15). The complete R-loop or RNA-RNA hybrid formation transitions the surveillance complex into an interference complex that ultimately cleaves the invading element (159). Within this unified theme, several differences exist in the interference mechanism ranging from recruitment of a Cas effector nuclease to the interference complex for target destruction *versus* conformational changes in the interference complex to activate target destruction. These different mechanisms of interference are discussed below.

### Class 1 systems

The Class 1 systems use a multi-protein helical assembly to degrade the target and, in certain cases, elicit collateral cleavage of nucleic acids irrespective of whether they belong to the host or the invader. The mechanisms used by the different types, and in some cases between the different subtypes, vary widely. Under Class 1, the type I systems and certain type IV subtypes target and destroy double-stranded (ds) DNA, whereas type III systems target and destroy RNA transcripts and single-stranded DNA (ssDNA).

#### Target DNA recognition by the type I systems

**Figure 3:**
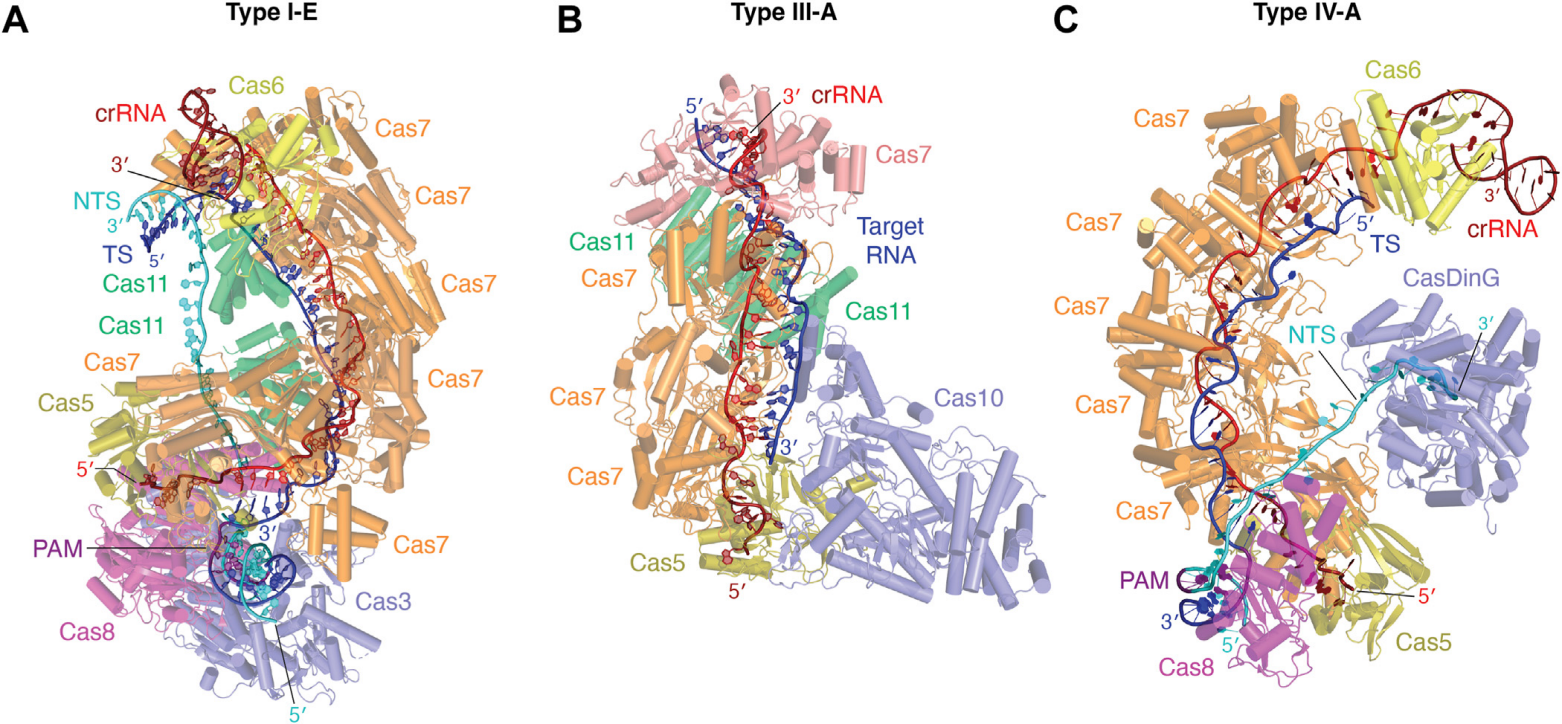
**Structures of the Class 1 interference complexes.** A single subunit of Cas5 and multiple subunits of Cas7 form the major filament in all Class 1 interference complexes. *A*, in type I-E Cascade from *Escherichia coli* (PDB ID 6C66) (126), the 3′-stem-loop of the crRNA is capped by Cas6 and the 5′-handle is capped by Cas5. The minor filament is composed of Cas11s and Cas8. Cas3 loads onto a bulge in the NTS through its interactions with Cas8. *B*, the type III-A Csm complex from *Thermococcus onnurineus* (PDB ID 6MUS) (108) is shown. The minor filament is composed of Cas11s and a large Cas10 subunit. *C*, the type IV-A system from *Pseudomonas aeruginosa* (PDB ID 7XG3) (98) has a helical structure similar to other Class 1 systems but lacks the minor filament. In this particular system, Cas5 covers the 5′-repeat of the crRNA and its 3′-stem-loop is capped with Cas6. CasDinG is shown bound to the nicked NTS. Figures were prepared using PyMol (369).

The mechanism of type I DNA recognition discussed in this section is based on the extensively studied type I-E system. The flipping of the sixth nt, which occurs upon binding of Cas7 to crRNA, creates a periodic pattern of 5 nt of accessible crRNA for base pairing with the target DNA (105). The target DNA is located through sequence-specific recognition of PAM by the Cas8 subunit. It is proposed that the type I PAM recognition occurs through minor groove interactions that account for the allowed promiscuity in PAM identification by this system (160, 161). After PAM recognition, Cas8 initiates unwinding of the dsDNA, leading to the formation of an R-loop (162). Positions 1 to 5 and 7 to 8 of the crRNA, flanking the PAM, are designated as the seed region for the type I Cascade complex, and complementarity between the crRNA and target DNA in the seed region is essential for R-loop formation (Figs. 1*A* and 3*A*) (83, 160) and fidelity of DNA cleavage. Once a stable R-loop covering the entire guide region is formed, the Cas11 subunits lock the TS DNA and part of the NTS DNA segments on opposite sides of the protein. The locking of the NTS DNA triggers the formation of a protruding “bulge” towards the 5′-end of the NTS and signals *trans*-recruitment of Cas3 in types I-B (110), I-C (113), I-E (126, 160), and I-F systems (107). Once recruited, a protruding loop from the Cas3 HD nuclease domain establishes interaction with the exposed groove of the Cascade, stabilizing the Cascade-Cas3 association (163) (Fig. 3*A*).

Contrary to the type I-E system, in types I-A (109), I-D (111), and I-G (115) systems, an autoinhibited Cas3 is an integral part of the surveillance complex that is essential for target DNA recognition and R-loop formation. In these systems, Cas3 exists in an inactive conformation until PAM-specific identification of target DNA and R-loop formation occur (164). This modified scheme prevents non-specific cleavage of DNA even though Cas3 exists as part of the surveillance complex. Another difference is in the domain organization of Cas3. In certain type I subtypes, Cas3 is expressed as a single polypeptide containing an HD nuclease and an SF2 helicase domain (*e.g.*, types I-B, I-C, I-E, and I-F), whereas in certain others, the two domains are expressed as two independent proteins, Cas3′ (SF2 nuclease) and Cas3″ (HD nuclease) (*e.g.*, types I-A, I-D and I-G, Table 2).

#### Target DNA degradation by the type I systems

Upon recruitment of Cas3, its HD nuclease domain introduces a nick in the NTS bulge, which triggers the helicase activity of the SF2 domain (17, 164, 165). The helicase activity unwinds the dsDNA upstream of the PAM, followed by reeling of ssDNA into the HD domain, which causes processive degradation of long stretches of both the NTS and TS DNAs (18, 109, 125, 166, 167, 168, 169). There are variations in the overall mechanism between the different type I subtypes (Table 2). Questions remain regarding how Cas3 elicits long-range processive DNA degradation. Two different models have been proposed in this context. One model suggests that post-nicking, Cas3 translocates along the DNA, independent of the surveillance complex, while the other model proposes that Cas3 stays associated with the Cascade and reels in and unwinds DNA using its helicase domain (169). It has been proposed that *cis*-cleavage of NTS and *trans*-cleavage of TS by the HD domain degrades DNA in the type I system (167). Further studies are essential to decipher the DNA cleavage mechanism of Cas3.

#### Target RNA recognition by the type III systems

Type III systems recognize RNA sequences complementary to their crRNA (19, 101, 108, 170, 171) (Fig. 1*B*). They may target both free transcripts, as well as nascent transcripts in the vicinity of a transcription elongation complex (TEC) (86, 172). The binding occurs in the vicinity of the TEC, where the two strands of the DNA remain unwound by the RNA polymerase (87, 173) (Fig. 1*B*). However, neither pull-down assays nor structural analysis have established any direct interaction between the RNA polymerase and the Csm/Cmr surveillance complex (173). In contrast to the PAM-mediated distinction of foreign DNA in types I/II/V, type III systems use a PFS (protospacer flanking sequence) to discriminate between self and non-self RNA (80, 81, 82, 108, 133, 174). While PAM-recognition requires sequence-specific interaction between the PAM of target DNA and a specific protein or a protein domain of the interference complex, PFS-recognition is based on the absence of complementarity at the 5′-end of the crRNA (called tag) and 3′-end of the target RNA (called anti-tag) (80, 174) (Fig. 1*B*). The type III system is unique since two components in the surveillance/interference complex, Cas7 and Cas10, are each capable of interference. Cas7 can cleave complementary RNA targets without fulfilling the PFS requirement, while the absence of base pairing between the tag:anti-tag (PFS) regions of the RNAs is essential to activate Cas10 for downstream activities (108, 133, 175). The seed requirements for type III are beginning to be identified and it is proposed that the 3′-end of crRNA is important for Cas7 (176, 177), while the 5′-end is essential for Cas10 activity (175, 178). There is also a study that reported that mismatches between the target and the guide were tolerated in a type III-A system (179). Comparison of the surveillance and interference complexes of the type III-B system from *Thermus thermophilus* shows the rotation of the non-catalytic Cas7 and Cas10 on either end of the helical filament, which in turn engender a slight rotation of the Cas11 subunits. The movement of Cas11 subunits exposes the guide region of the crRNA to pair with the target RNA (132). Thus, the complex accommodates the target within the surveillance complex assembly with only minimal conformational changes, which is similar to type III-A (108) and type I (Target DNA recognition by the type I systems) systems.

#### RNA and DNA degradation by the type III systems

The type III systems can execute three different cleavage pathways: Cas7-mediated sequence-specific target RNA cleavage (19, 170, 180), Cas10-mediated ssDNA cleavage (86, 87), and cyclic oligoadenylate (cOA)-signaled and ancillary protein-mediated non-specific nucleic acid or protein cleavage (29, 181, 182).

Since Cas7 activity is PFS-independent (86), the pairing of target RNA with the guide region of the crRNA triggers periodic cleavage at every sixth nt by the catalytic Cas7-like proteins of the major filament (19, 101, 108, 170, 171) (Figs. 1*B* and 3*B*). For activation of Cas10, there are two prerequisites: first, around 5 nt-long mismatch between the 5′-crRNA-tag and the 3′-target RNA anti-tag (PFS), and second, ∼12 nt-long complementarity between the crRNA and the target RNA flanking the crRNA-tag (81, 85, 87, 108, 112, 175, 183) (Figs. 1*B* and 3*B*). The PFS-mediated Cas10 activation occurs through specific protein conformational changes enabling, independently, ssDNA cleavage by the HD-nuclease domain and production of cOA by the Palm domains (81, 86, 87, 175, 182, 183). While the Cas7-mediated target RNA cleavage is independent of Cas10 activation (85, 129, 184), the cleavage of target RNA by Cas7 releases the cleaved RNA, which resets the ssDNase and cOA production activities of Cas10 (81, 87, 183, 185).

The cOAs are small cyclic RNA second messengers possessing varying numbers of adenosine monophosphate units that change the size of the adenylate rings (cA_3-6_) (182, 186). While both Palm domains of Cas10 bind to ATP, the active site for cyclization (a DD motif) is inserted into the Palm2 domain (187, 188, 189). The cOA ligand binds to the conserved CARF (CRISPR-associated Rossman fold) domain of ancillary proteins such as Csm6/Csx1/Can1/Can2/Card1 (Fig. 1*C*), activating non-specific cleavage of nucleic acids and causing cell dormancy or death (182, 186, 187, 190). Csm6 and Csx1, upon activation by cA_4/6_ and cA_4_, respectively, impart HEPN-mediated non-specific ssRNA cleavage (191, 192). cA_4_-bound Can1 (193) cleaves dsDNA plasmids, whereas Card1 (194) and Can2 (195) degrade dsDNA, ssRNA, and ssDNA. The NucC endonuclease (lacks the CARF domain) dimerizes upon binding to cA_3_ and activates a downstream anti-phage signaling system that destroys the host chromosome, causing cell death. Interestingly, cOA signaling was observed to induce cooperative phage clearance by type I and type III systems (196). In this case, cA_4_, produced by the type III system, binds to a transcription factor, Csa3, produced from the type I-A locus, which regulates several CRISPR and DNA repair genes (196, 197, 198, 199). The cOA signaling process is reset through the degradation of cOA molecules by ring nuclease activity of proteins such as, AcrIII-1 (Anti-CRISPR III-1), Csx3 or Csm6 (181, 200, 201, 202, 203, 204, 205).

Recently, a new type III subtype (type III-E) with several unique features has been reported. Unlike all Class 1 subtypes that contain multiple protein subunits in the helical assembly, the type III-E system possesses a multi-domain single effector Cas7-Cas11 protein (also called gRAMP, giant repeat-associated mysterious protein) (28). The gRAMP protein has right-handed helical features and consists of four Cas7-like domains, one Cas11-like domain, and a big insertion domain (BID) (15, 29, 206). These systems lack the larger Cas10 subunit and instead recruit Csx29 which is a caspase-like protein with an N terminal tetratricopeptide repeat (TPR) also called TPR-CHAT (CHAT: Caspase HetF associated with TPRs). The TPR-CHAT interacts with the Cas7-like domain of the gRAMP at the 5′-handle of the crRNA forming an effector complex (206, 207), known as Craspase (CRISPR-guided caspase) (15, 208). Similar to Cas10 found in other type III systems, activation of TPR-CHAT depends on non-complementary PFS recognition. It is proposed that upon activation, the TPR-CHAT binds to a complex composed of Csx30, Csx31, and RpoE transcription factor (209). Recent studies have shown that site-specific cleavage of Csx30, a protein produced from the Cas operon (29, 208), by TRP-CHAT activates antiviral immunity (208, 209, 210, 211). However, the molecular mechanism of defense is yet to be determined.

One of the outstanding questions in type III systems is what type of DNA is cleaved by Cas10. While an earlier study reported that Cas10 cleaves the ssDNA region near the TEC (86), there are reports of Cas10 cleaving DNA that is not part of a transcription bubble (for example, free ssDNA and exposed ssDNA in a dsDNA) (172, 173). Cas10’s ssDNA cleavage activity has been related to increased cellular mutation frequency, which was abolished with an inactive Cas10 (212). Further studies are needed to determine how exactly Cas10 targets ssDNA inside cells. Other interesting questions will be to understand how distinct cOA molecules activate a suite of ancillary proteins to induce dormancy or cell death following type III immune response and how these diverse activities are coordinated.

#### Target DNA recognition by the type IV systems

The mechanisms of type IV CRISPR systems are slowly emerging. Currently, characterized type IV systems have shown PAM-dependent target DNA recognition mediated by Cas8. The type IV-A surveillance complex contains only the major filament, similar to the type I-F system (98, 213). Similar to type I systems, upon PAM recognition, the Cas8 subunit initiates unwinding of the dsDNA. Positions 1 to 5 and 7 to 8 of the crRNA guide region, flanking the PAM, constitute the seed sequence. Following R-loop formation, due to the lack of Cas11 subunits, the TS of the target DNA is stabilized by the Cas7 subunits, whereas the displaced NTS is stabilized by the Cas8 subunit (98). While the type I-F surveillance complex elongates when it forms the interference complex (Table 2), the type IV-A interference complex undergoes a ∼10 Å contraction (98). The structure available for the IV-B subtype is incomplete because Cas5 and Cas8 are not present in the complex (136).

#### Target DNA degradation by the type IV systems

Type IV-A has been related to plasmid clearance that is dependent on the helicase activity of CRISPR-associated Damage inducible gene G (CasDinG) (104, 214, 215, 216) (Fig. 3*C*). CasDinG is recruited to the single-stranded region of the R-loop present in the helical filamentous complex. CasDinG is composed of an SF2 helicase core (consisting of two RecA-like helicase domains), and three accessory domains (N-terminal, arch, and vFeS) (98, 216). CasDinG unwinds dsDNA in an ATP- and metal-dependent manner (mentioned in Type IV-A DNA helicase: CasDinG) similar to the type I Cas3 subunit (98, 216). Unlike Cas7 subunits in other Class 1 systems, Cas7 in type IV-A contains an additional forefinger that recruits CasDinG (Fig. 1*C*). These forefingers are hypothesized to facilitate the sliding of CasDinG to unwind the dsDNA in a 5′→3′ direction (opposite to Cas3’s 3′→5′ unwinding). The ssDNA-ATP-dependent helicase activity of CasDinG has been experimentally confirmed, with the proposal that a cellular nuclease may degrade the ssDNA generated by CasDinG (98). A recent study has identified a rare group of type IV-A system from metagenomic data mining. CasDinG of this system has an inserted HNH domain that was demonstrated to have nuclease activity (26). The colocalization of distinct proteins in the type IV CRISPR locus for each subtype (*i.e.*, the signature protein) has led to the proposal that each subtype may direct distinct biological functions (217).

### Class 2 systems

Unlike the Class 1 systems, Class 2 systems possess multi-domain, single-effector nucleases that eliminate the need for the external recruitment of nuclease proteins. Instead, allosteric activation of Class 2 Cas proteins for target cleavage is initiated by structural rearrangements triggered by crRNA-DNA/RNA hybridization (15).

#### Target DNA recognition by the type II systems

**Figure 4:**
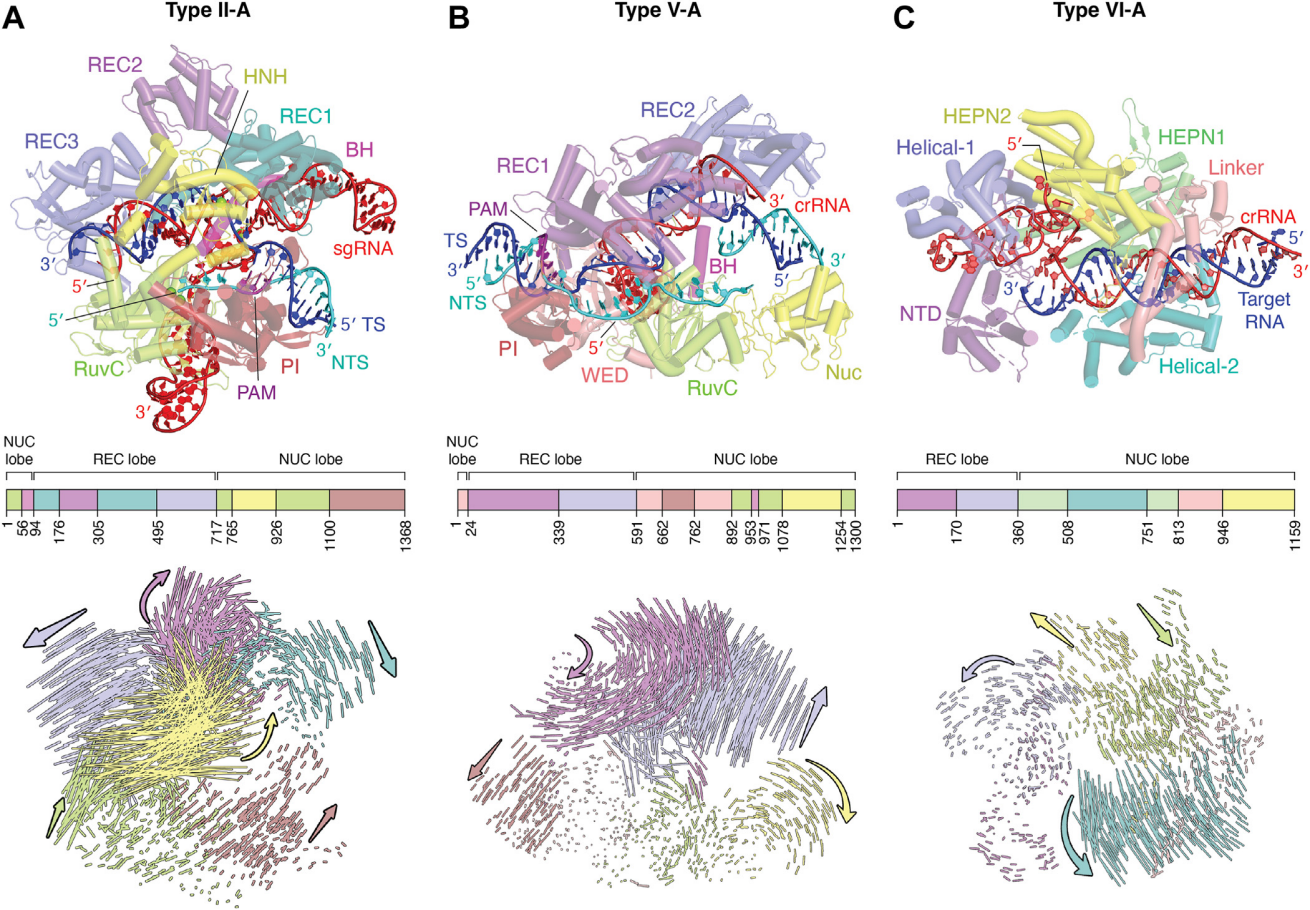
**Structure of Class 2 interference complex (top) and the movement of the protein domains upon binding of the surveillance complex to the target nucleic acid (*bottom*).** The *lines* represent the magnitude of movement of the alpha carbon for each amino acid, and the arrows indicate the direction of movement of each domain from surveillance to the interference state. *A*, structure of the SpCas9 interference complex (PDB ID 7Z4J) (370). When SpCas9 surveillance complex (PDB ID 4ZT0) (146) transitions to the interference complex (PDB ID 7Z4J), conformational changes occur in the REC and NUC lobes. *B*, structure of FnCas12a interference complex (PDB ID 6I1K) (236). As the FnCas12a surveillance complex (PDB ID 5NG6) (154) transitions to the interference complex (PDB ID 6I1K), major conformational changes occur in the REC lobe and minor changes occur in the NUC lobe. A part of the NTS (*cyan*) is not visible in this structure. *C*, structure of Cas13a interference complex (PDB ID 5XWP) (157). Transitioning from the surveillance complex (PDB ID 5XWY) (157) to the interference complex (PDB ID 5XWP), results in major conformational changes in the NUC lobe. Within each panel, the middle scheme shows the amino acid range for each domain and the structure is color-coded based on this scheme. Figures were prepared using PyMol (369).

The structural rearrangements that occur upon surveillance complex formation, expose a pre-ordered 10 to 12 nt-long “seed” region in the crRNA guide that is crucial for DNA interrogation and subsequent R-loop formation (77, 218, 219). The structural changes of the Cas9-sgRNA complex also enable the ordering of two crucial arginine residues within the PAM-interacting (PI) domain to sequence-specifically identify the PAM sequence in the NTS of the target DNA (146, 148, 149). The cognate PAM binding initiates DNA melting and strand switching that is facilitated by a phosphate lock loop in the PI domain. These changes introduce a kink in the TS of the target DNA to enable its base pairing with the seed region of the crRNA flanking the PAM, thereby producing a single-stranded NTS and initializing the R-loop formation (74, 77, 220, 221). The RNA-DNA hybrid runs through the central channel, while the displaced NTS threads in between the RuvC and HNH domains through the NUC lobe (Fig. 4*A*).

#### Target DNA degradation by the type II systems

Post-DNA melting, the HNH domain acts as a conformational checkpoint in response to the length of the RNA-DNA base pairing that is critical to ensure the fidelity of DNA cleavage beyond the stable R-loop formation (222). Specifically, once the R-loop is 17 nt long, the HNH domain undergoes a 180º reorientation through the stabilization of two linker helices (L1 and L2) that places the HNH active site closer to the scissile phosphate of the TS strand (Fig. 4*A*) (223). The movement of the HNH domain also removes the steric hindrance that prevents RuvC from accessing the NTS (138, 222, 223, 224). Once HNH and RuvC are placed at their respective scissile phosphates, they can independently cleave the respective DNA strands. Several kinetic studies have reported a faster rate of cleavage for HNH compared to RuvC, a feature that has been attributed to extra conformational changes needed for the RuvC domain to reach a cleavage competent state and/or for resolution of the R-loop (225, 226). Interestingly, kinetic studies that measured cleavage kinetics devoid of conformational requirements reported identical rate constants for both endonuclease domains (227). Divalent metal-ion recruitment and NTS DNA positioning are proposed to be essential for stabilizing the closed conformation essential for DNA cleavage (228). This model also explains the trapping of the complex in an intermediate state incapable of DNA cleavage when mismatches are present in the DNA (222, 229, 230). Cas9’s DNA cleavage introduces two single-stranded scissions, one on the TS and the other on the NTS, by the HNH and RuvC endonuclease domains, respectively, creating mostly a blunt-ended cleavage product (77, 138, 229) (Fig. 2*A*).

#### Target DNA recognition by the type V systems

Cas12a, an exemplar of the type V systems, requires only crRNA to target dsDNA that contains a PAM at the 5′ end of the NTS (Fig. 2*B*) (23). The pre-crRNA binding is mediated by sequence and shape-specific recognition of the repeat region, which exposes a pre-ordered seed segment to enable locating the target DNA. The target DNA location is initiated by the identification of the cognate PAM by the Wedge and PI domains of Cas12a, which causes a bend in the DNA. This step triggers unwinding of the dsDNA and results in the hybridization of crRNA-TS and displacement of the single-stranded NTS, forming the R-loop (154). Base pairing starts between the seed region of the crRNA and PAM-proximal nucleotides of the TS and extends toward PAM-distal nucleotides to form the R-loop. Compared to Cas9, R-loop is more reversible in Cas12a which leads to more mismatch sensitivity in Cas12a (231, 232). In Cas12a, the REC lobe undergoes significant conformational changes in the interference complex, while the NUC lobe experiences alterations only in the Nuc domain (Fig. 4*B*). Similar to Cas9, conformational changes in the bridge helix of Cas12a are important for its activity (233, 234, 235).

#### Target DNA degradation by the type V systems

Unlike Cas9, Cas12a uses RuvC to cleave both strands of the DNA and exhibits an order with NTS cleavage being a prerequisite for TS cleavage (236, 237). The cleavage generates a staggered dsDNA break (Fig. 2*B*), and this activity is referred to as *cis*-cleavage (23). A distinct feature of all type V systems is the presence of a “lid” covering the active site of RuvC that undergoes a loop-to-helical transition, which is believed to be important for the RuvC domain to reach the DNA cleavage-competent state (mechanism discussed in Type V nuclease domains: crRNA processing site and RuvC). However, details of how the lid promotes NTS and/or TS cleavage are not completely known (235, 238, 239, 240, 241). Even though RuvC is very rigid with minor changes between the surveillance and interference complexes, the movements of the Nuc domain and REC2 domain are critical in the regulation of dsDNA cleavage by the RuvC nuclease domain (242) (Fig. 4*B*). While NTS cleavage is exclusively performed by RuvC, TS cleavage needs assistance from the Nuc domain to produce a combined active site pocket made of RuvC and Nuc residues (140, 243, 244). The nicking of the NTS enables traversing of the TS as a ssDNA into the RuvC cleavage pocket with a 5′→3′ directionality. The extra nt of the TS (with respect to the cleavage position of the NTS) needed to reach the RuvC site results in staggered dsDNA break in most Cas12 subtypes (237, 245, 246). Another distinction from Cas9 is that Cas12a cleaves distal to the PAM and releases the distal product of *cis*-cleavage, leaving the RuvC domain exposed to nick dsDNA or degrade ssDNA in a non-specific manner (*trans*-cleavage) (236, 247, 248, 249, 250).

#### Target RNA recognition by the type VI systems

Type VI systems target and cleave single-stranded regions of an RNA that has complementarity with the guide region of the crRNA using their HEPN domains. Compared to type II and type V systems that use preordered and solvent-exposed seed regions, the seed region in the Cas13a binary complex is disordered and located in the middle of the guide region of the crRNA (251), resulting in an unfavorable energy barrier for crRNA and target RNA duplex formation (139). Overcoming this barrier requires complementarity between crRNA and target RNA covering the entire protospacer region. Thus, Cas13a has a lower tolerance for mismatches compared to the interference nucleases of type II and type V systems (30, 251). In addition to target RNA complementarity, some Cas13 systems require (albeit variably) the PFS for activation of RNA cleavage. While certain Cas13a variants need PFS (30), certain other Cas13a variants (252) and Cas13d lack a stringent PFS requirement (253, 254, 255). Significant conformational changes occur in the Helical-2 domain of Cas13a upon binding to crRNA to accommodate the crRNA-target RNA duplex (139, 157, 253). Target RNA binding induces further conformational changes in the Helical-2 domain and rotation of the HEPN1 domain towards the crRNA-target RNA hybrid (Fig. 4*C*), which produces an RNA cleavage site in between the HEPN1 and HEPN2 domains (157). This conformational change occurs only when the PFS requirement is met (*i.e.*, no pairing between the tag and the anti-tag regions) in those subtypes that follows the PFS requirement. If the tag and anti-tag are base-paired, it serves as a negative determinant as it induces conformational changes in both the repeat region of the crRNA and the NUC lobe of Cas13a, which prevents assembly of the HEPN catalytic pocket (256, 257).

#### Target RNA degradation by type VI systems

Cas13a possesses two distinct RNA cleavage sites, one for processing the single pre-crRNA and another for cleaving target RNA (139). Cas13a cleaves the 5′-end of the pre-crRNA within the repeat region using a composite active pocket formed at the interface of the Helical-1 and HEPN2 domains, and it remains bound to form the surveillance complex. Based on the Cas13a variant, the catalytic residues can be in the HEPN2 or Helical-1 domain (139, 157, 258). When a target RNA is located, a new active site pocket is assembled between the interface of the two HEPN domains and is primed for cleavage of exposed ssRNAs (Fig. 2*D*) (139, 157). The active site is located at the exterior of the bilobed protein (139). The cleavage can be *cis*-cleavage (*i.e.*, Cas13 cleaves a single-stranded region extending from the target RNA that is bound to the crRNA) or collateral/*trans*- cleavage (*i.e.*, cleavage of a target RNA extending from a bystander Cas13 ternary complex or that of non-specific ssRNA in the vicinity) (139, 259, 260).

## Catalytic mechanisms related to CRISPR-Cas interference

The highly diverse CRISPR-Cas systems utilize similar nucleic acid cleavage mechanisms (15, 16, 104). Based on the type of end products, the Cas proteins can be broadly classified into divalent metal-ion-dependent and -independent reactions (261). Most of the subtypes cleaving DNA substrates use a divalent metal-ion-dependent general acid-base catalytic mechanism (type I: Cas3; type III: Cas10; type II: Cas9; type V: Cas12) that generates a 5′-phosphate and a 3′-hydroxyl as products (77, 154, 261). Cas proteins that cleave ssRNA use either an HEPN domain (type III: ancillary proteins, type VI: Cas13) or an RRM domain (Class1: Cas6, type III: Cas7) to trigger metal-independent general acid-base catalysis rendering products with a 5′-hydroxyl and a 2′,3′-cyclic phosphate (19, 258, 261).

### Class 1 nucleases and helicases

#### Class1 crRNA biogenesis: Cas6

The crRNA biogenesis reaction by Cas6 is similar to the mechanisms of archaeal tRNA splicing endonucleases and RNase A (68, 262, 263, 264). In these systems, a conserved Tyr/His serves as the general base, a His residue is the general acid, and a Lys residue stabilizes the transition state (265, 266, 267, 268). The intermediate crRNA generated through this process has 5′-hydroxyl and 2′,3′-cyclic phosphate ends (68, 266).

#### Type I defense nuclease: Cas3

**Figure 5:**
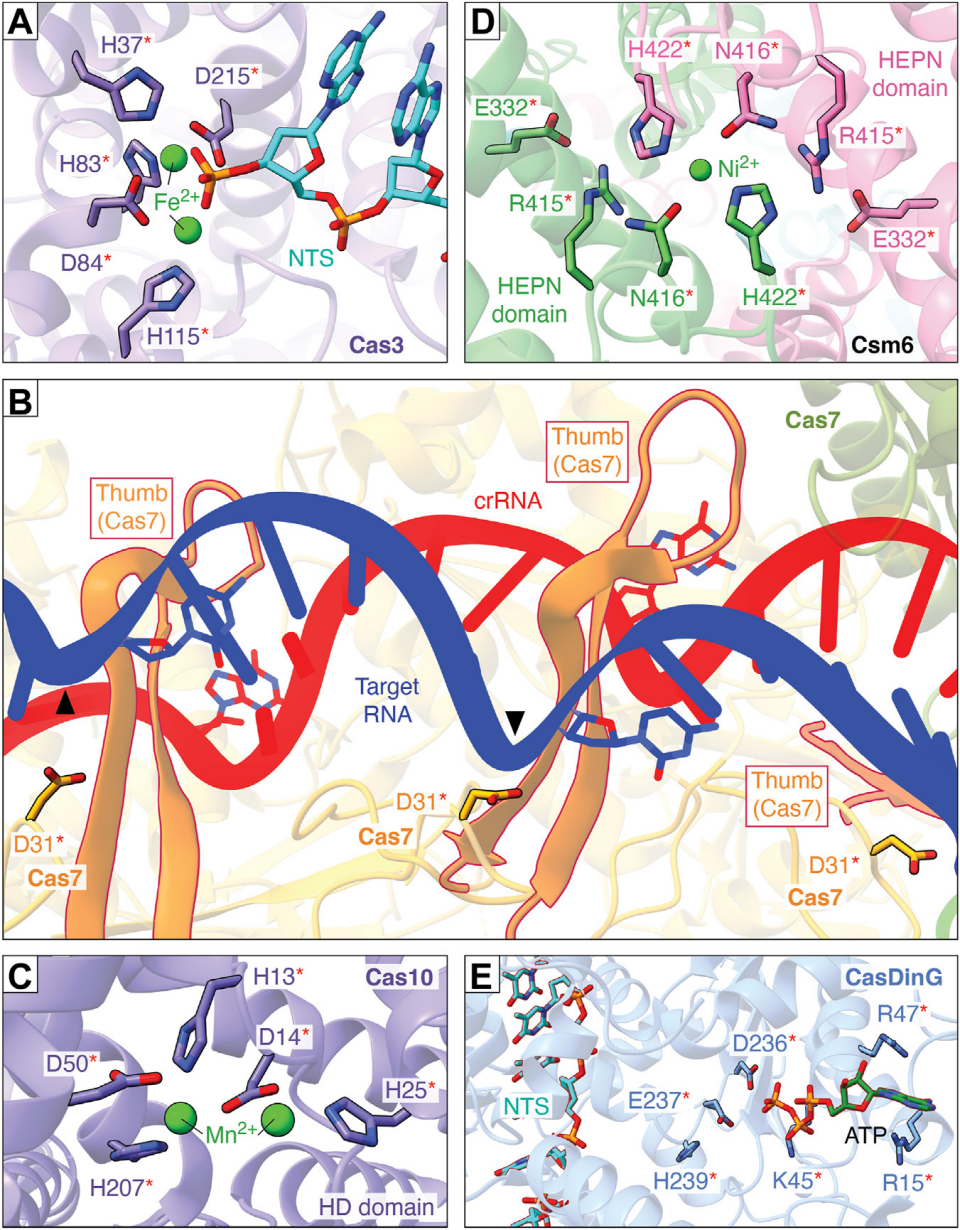
**A close-up view of the active site of selected Class I Cas proteins.** Red asterisks show catalytic residues. *A*, the active site of Cas3 HD nuclease domain from a type I-E system with two Fe^2+^ ions and the conserved His and Asp residues are shown (PBD ID 4QQW) (164). *B*, the thumb region of Cas7 in type III systems inserts into the crRNA-target RNA duplex at every sixth nucleotide, positioning a conserved Asp in the catalytic loop near the scissile phosphate, enabling a periodic cleavage in the RNA backbone (PDB ID 3X1L, type III-B) (171). *Black triangles* indicate the cleavage sites. *C*, the active site of the Cas10 HD domain belonging to a type III-B system with two transition metal-ions (Mn^2+^) and the conserved His and Asp residues are shown (PDB ID 4W8Y) (371). *D*, the HEPN active site of Csm6 is organized at the dimer interface (PDB ID 5FSH, type III-A) (372). The Ni^2+^ ion captured by the conserved catalytic triad was proposed to be an artifact from crystallization. *E*, the type IV-A CasDinG with the important residues of the ATP binding pocket marked with an *asterisk* (PDB ID 7XF0) (98). We positioned NTS-DNA into 7XF0 based on its position in the CasDinG-ssDNA complex (PDB ID 6FWR) (373). Figures were prepared using ChimeraX (374).

Cas3 contains an N-terminal HD nuclease and a C-terminal SF2 helicase domain for coordinated ssDNA cleavage and DNA unwinding, respectively (3, 269). The conserved His and Asp residues in the HD domain coordinate two divalent transition metal ions (*e.g.*, Fe^2+^, Ni^2+^, Mn^2+^, Co^2+^ and Zn^2+^) at the ssDNA cleavage site that is indispensable for the two metal-ion-mediated DNA cleavage (Fig. 5*A*) (17, 164, 165, 168, 270, 271). Cas3’s ATP-dependent SF2 helicase mechanism is similar to other well-understood DexD/H-type SF2 helicases (272, 273, 274). Similar to the SF2 helicases, Cas3 helicase domain possesses helicase motifs such as Q, I, II, and VI that bind and hydrolyze ATP, enabling DNA unwinding (17, 275). In Cas3, ATP hydrolysis is Mg^2+^-dependent, and the protein possesses the typical Walker A and B motifs. While the inactivation of the ATPase domain compromises the helicase activity of the SF2 domain, it does not affect the nuclease activity of the HD domain (17, 164, 168, 275).

#### Type III defense nucleases: Cas7, Cas10, and ancillary proteins

The Cas7-like proteins in type III systems are unique compared to other Class 1 Cas7s because they can cleave RNA. The target RNA cleavage by Cas7 in type III systems is proposed to be a divalent metal-ion-independent reaction since it produces 5′-hydroxyl and 2′,3′-cyclic phosphate ends (19, 171, 183). However, studies have shown that the reaction cannot proceed without a divalent metal ion necessitating further in-depth characterization (19, 101, 171). In Cas7, deprotonation of the 2′-hydroxyl of the target RNA is carried out by an unknown general base, followed by the nucleophilic attack of the 2′-oxyanion on the scissile phosphate. An invariant Asp residue serves as the general acid (Fig. 5*B*) (276).

The HD nuclease of Cas10 is proposed to use acid-base catalysis for ssDNA cleavage, with His acting as the base and Asp acting as the acid (Fig. 5*C*) (133). While the HD domains of Cas10 and Cas3 are evolutionarily distinct, they both require a two-divalent transition metal-ion-mediated DNA cleavage facilitated by Ni^2+^,Co^2+^, or Mn^2+^, instead of Mg^2+^, to activate ssDNA cleavage (81, 87, 172, 173, 183, 277, 278).

The divalent metal-ion-independent RNA cleavage activity of ancillary proteins Csm6 (191) and Csx1 (192) was impaired by substitutions of conserved Arg and His residues of the HEPN domain, indicating the importance of these residues in ssRNA catalysis (Fig. 5*D*) (279). It has been proposed that His can act as a general acid/base and Arg stabilizes the intermediate state (192).

#### Type IV-A DNA helicase: CasDinG

The SF2 helicase core of CasDinG uses 5′→3′ DNA translocase activity to unwind dsDNA in an ATP-dependent manner. Residues such as Arg, Lys, Asp, and Glu play a crucial role in stabilizing the ATP-binding pocket of CasDinG (Fig. 5*E*). Studies have demonstrated that divalent metal ions such as Mg^2+^, Ni^2+^, Ca^2+^, or Co^2+^ are necessary for the helicase activity of CasDinG. Deletion studies have shown that the N-terminal accessory domain is necessary for plasmid clearance (216), however, the exact events leading to plasmid clearance remain unknown.

### Class 2 nucleases

#### Type II nuclease domains: HNH and RuvC

**Figure 6:**
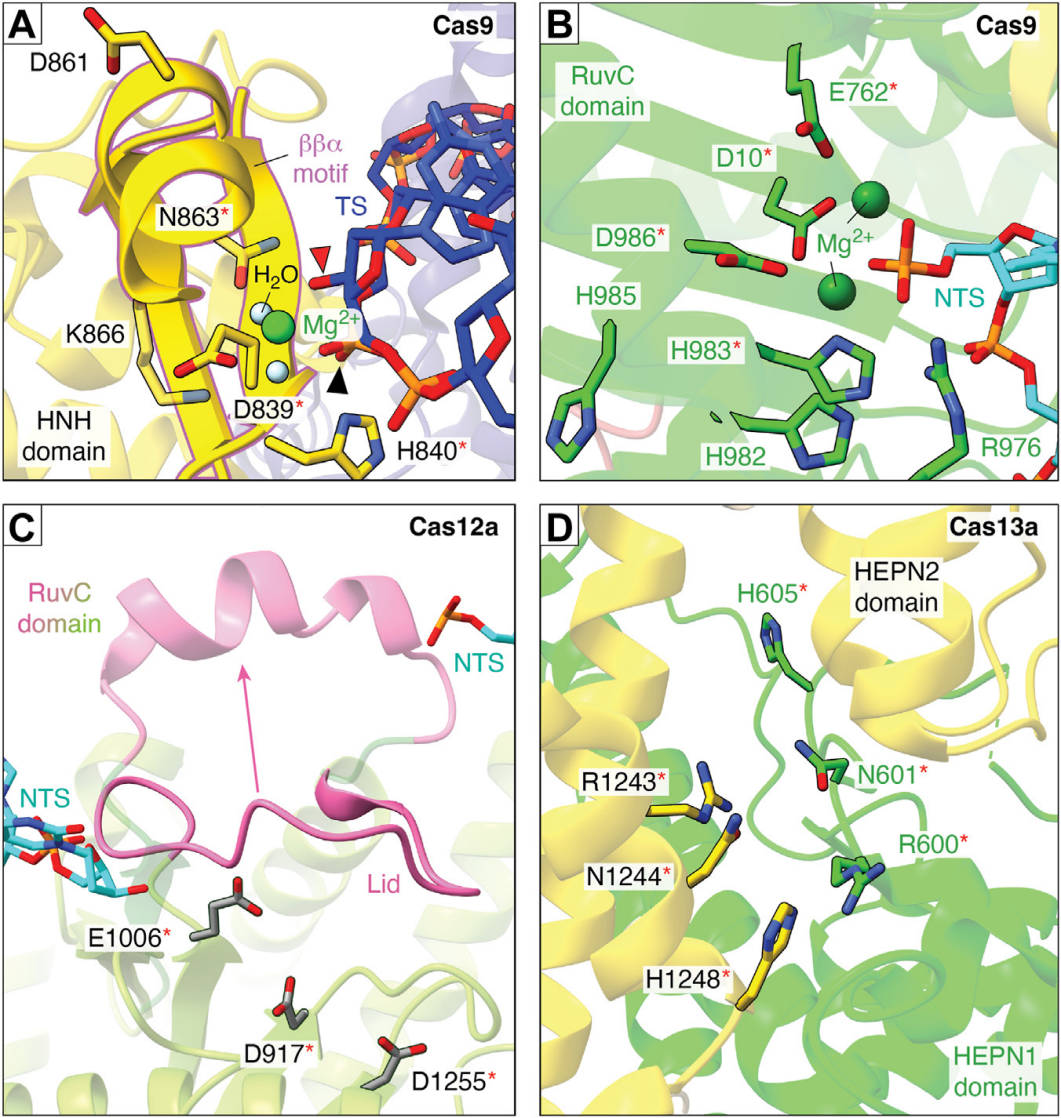
**A close-up view of the active site of Class 2 Cas nucleases.***Red asterisks* show catalytic residues. *A*, HNH domain of Cas9 (PDB ID 7Z4J, type II-A) (370) consists of a ββα motif and cleaves the TS DNA by a one-divalent metal-ion-mediated catalysis. *Red* and *black triangles*, respectively, show 3′hydroxyl and 5′-phosphate ends after TS cleavage. *B*, RuvC domain of Cas9 (PDB ID 7Z4J, type II-A) cleaves NTS DNA by a two-divalent metal-ion-mediated catalysis. Only part of the NTS is visible since it is disordered in the structure. *C*, Cas12a (PDB ID 6I1K, type V-A) (236) uses a RuvC active site to cleave both NTS and TS of the DNA. RuvC active site is blocked by a “lid” (*pink*), which transforms into an α helix (PDB ID 6GTG) (237) upon DNA binding, providing access for ssDNA to reach the catalytic site (divalent metal-ions and the rest of the NTS DNA were not captured in both the Cas12a structures). *D*, the conserved catalytic residues (RX_4_H) of Cas13a′s (PDB ID 5WLH, type VI-A) (295) HEPN1 and HEPN2 domains are positioned for a divalent metal-independent RNA cleavage. RNA was not captured in the structure. Figures were prepared using ChimeraX (374).

Cas9 uses two separate endonuclease domains, HNH and RuvC, to cleave the TS and NTS, respectively, of a dsDNA to introduce a dsDNA break and generates products with 5′-phosphate and 3′-hydroxyl ends (76, 77, 261, 280, 281). The HNH domain contains a ββα-metal catalytic core and cleaves TS DNA using a single divalent metal-ion-mediated catalysis (138, 261, 282, 283, 284). Based on structural and mutational studies of Cas9’s HNH domain, the catalytic triad is composed of Asp, His, and Asn (Fig. 6*A*) (77, 138). His acts as a general base activating a water molecule for the nucleophilic attack on the scissile phosphate (138). The Asp and Asn coordinate the divalent metal-ion that stabilizes the transition state.

The RuvC domain of Cas9 possesses an RNase H fold and cleaves the NTS DNA using a two-divalent metal-ion-dependent catalytic mechanism (Fig. 6*B*). Structural and mutational experiments (in *S. pyogenes*) have shown that RuvC has a conserved DDE motif and a catalytic His residue that is proposed to be the general base required for activating a water molecule and promoting DNA cleavage (228, 261, 285). Other studies have proposed that other His residues in the active site of *S. pyogenes* RuvC can act as general bases (286), pointing to the flexibility in the choice of the His residue. Two invariant Asp residues (D10 and D986 in *S. pyogenes* Cas9) coordinate the Mg^2+^ ions, which are essential for transition state stabilization (286, 287). Computational studies have predicted that the sidechain interaction between an Arg of the RuvC domain and a Glu of the HNH domain is involved in coordinating the dsDNA cleavage. The proposed mechanism is that the movement of HNH towards the TS DNA leaves Arg free to interact with the NTS and to stabilize the RuvC catalytic state (288, 289).

#### Type V nuclease domains: crRNA processing site and RuvC

Cas12a possesses both pre-crRNA processing and DNA cleavage activities, which are both mediated by general acid-base catalysis. The active site for pre-crRNA processing is located in the Wedge domain (Fig. 4*B*). Studies of *Francisella novicida* Cas12a (FnCas12a) showed residues H843 (acid), K852 (transition state stabilization), and K869 (base) (290) as the key players that are proximal to the 5′-flanking region of the repeat’s stem-loop (23, 291). Although a divalent metal-ion is required for the binding of crRNA to Cas12a, it is not required for RNA cleavage (154). The reaction proceeds by a nucleophilic attack of the 2′-hydroxyl group of the upstream nucleotide on the scissile phosphate of the pre-crRNA, creating 5′-hydroxyl and 2′,3′-cyclic phosphate groups.

The catalytic residues for DNA cleavage are located in the RuvC domain (D917, E1006, and D1255 in FnCas12a), which employs a two-divalent metal-ion mechanism for DNA cleavage (Fig. 6*C*) (23, 290). Although these catalytic residues belong to the RuvC domain, mutation of Nuc domain residue (*e.g.*, R1218 in FnCas12a) impairs TS cleavage due to the role of Nuc domain in DNA binding and active site-pocket formation (140, 154, 243, 244). Exceptions are present in diverse Cas12 families, for example, Cas12c, Cas12g, and Cas12j, that use the RuvC domain to cleave RNA and/or DNA (39, 155, 238, 292, 293). Cas12k associated with CRISPR-associated transposases (CAST) does not possess the ability to cleave RNA or DNA due to amino acid substitutions in the active site (36, 294) (Table 1).

#### Type VI nuclease domains: crRNA processing site and HEPN

Cas13 possesses two distinct active sites to process pre-crRNA and to cleave target RNA. The pre-crRNA processing utilizes a metal-independent acid-base catalysis, similar to Cas12a, and the active site is located at the interface of the Helical-1 and HEPN2 domains (Fig. 4*C*) (139, 157). Multiple residues have been proposed to participate in proton transfer, leading to the formation of a 2′,3′-cyclic phosphate and a ribose 5′-hydroxyl, with a Lys proposed to be the general base (139, 295). A divalent metal-ion contributes to pre-crRNA folding/binding, but not to crRNA cleavage (295).

The RNase active site for target RNA cleavage, including *cis*- and collateral cleavages, is located at the interface of the two HEPN domains (Fig. 4*C*) comprising two RX_4_H motifs, one each from HEPN1 (R600 and H605 in *Lachnospiricae bacterium* (Lba) Cas13a) and HEPN2 (R1243 and H1248 in LbaCas13a) (Fig. 6*D*). Substitution of any one of these His or Arg residues renders Cas13a inactive (139). Additionally, an Asn residue located next to the Arg of the RX_4_H motif (N601 in HEPN1 and N1244 in HEPN2 of LbaCas13a) is essential for RNA cleavage (139, 295). The target RNA cleavage by Cas13 is also metal-independent. The target RNA cleavage in Cas13 differs from single HEPN-containing enzymes such as Csx1 and Csm6 that dimerize to bring in two HEPN domains together to form the active site (139, 295).

## Relationship of CRISPR-Cas RNA cleaving enzymes with other RNases

The molecular mechanisms used in CRISPR-Cas systems are related to Argonautes, ribozymes, and other RNases (296, 297). The guiding principle of CRISPR-Cas systems, which is RNA-guided nucleic acid-targeting followed by repression or cleavage, is similar to eukaryotic RNA interference (RNAi) and prokaryotic Argonaute-based systems (296). Similar to Argonautes, most CRISPR systems use a pre-ordered RNA seed for target searching, which is a mechanism that has been conserved in both RNA- and DNA-targeting CRISPR systems (105, 146, 163, 298, 299). As mentioned previously, the length of the seed and how much of it is exposed in the surveillance complex vary between the different CRISPR types (139, 243, 251). Another similarity is the ruler-based cleavage mechanism conserved in both Argonautes and CRISPR systems, specifically the type III CRISPR systems (19). CRISPR systems have also been shown to regulate physiological functions such as CRISPR-Cas expression (300, 301), modulation of pathogenicity in *F. novicida* (302, 303), and several other roles using the RNA-guided nucleic acid repressing principle similar to RNAi (50).

As mentioned in cleavage mechanisms, most of the current knowledge supports a nucleic acid cleavage mechanism similar to RNases (261) and has an evolutionary relationship to HEPN domains found in RNA-cleaving toxin-antitoxin systems (304). Examples include Cas13, Csm6, and Csx1, which use a metal-independent, protein residue-mediated, acid-base catalysis for RNA cleavage similar to the HEPN-mediated RNA cleavage in eukaryotic RNases such as Ire1, RNase L, and RNase PNK (305). Other ancillary nucleases such as Card1 (194), Can1 (193), and Can2 (195) use divalent metal-ion-dependent DNA/RNA cleavage mechanism similar to RNase H (306). Interestingly, ribozymes possess distinct RNA cleavage mechanisms involving both divalent metal-ion-independent (307, 308, 309, 310) and -dependent [both one- (311, 312, 313) and two-metal ions) (314, 315, 316)] processes, similar to the diverse cleavage mechanisms seen in Cas enzymes (see Catalytic mechanisms related to CRISPR-Cas interference). The metal-independent 2′-O-*trans*-phosphorylation mechanism mediated by acid-base catalysis, which produces a 2′,3′-cyclic phosphate and a 5′-hydroxyl group, is present in several RNA-cleaving Cas enzymes and ancillary proteins (explained in Catalytic mechanisms related to CRISPR-Cas interference). These cleavage end products are different from those of Argonautes, which have 5′-phosphate and 3′-hydroxyl groups (317).

Similar to CRISPR-RNases adapting cleavage mechanisms from other RNA-cleaving systems (both protein- and RNA-based), DNA cleavage mechanisms of CRISPR-Cas systems are also present in other DNA metabolizing enzymes. Examples of this include the homing endonuclease mechanism of HNH in Cas9, RuvC endonuclease used to resolve Holliday junctions in Cas9 and Cas12, HD nuclease in Cas3 and Cas10, and the SF2 helicase in Cas3 and CasDinG (278, 318, 319, 320). Thus, there appears to be a mechanistic convergence between the CRISPR-Cas enzymes, RNases, and DNases based on the general acid-base catalysis mechanism used for cleaving the phosphodiester bond and the type of cleavage products. This cleavage mechanism and product formation is also conserved in ribozymes (261, 321). A recent study has discovered a naturally occurring hydrolytic endonucleolytic ribozyme (HYER) in Group II-C introns that uses a specific RNA sequence motif present in the ribozyme to sequence-specifically locate and cleave DNA (322). This appears to be another example of convergence between the CRISPR-Cas system and the ribozyme where the similarity extends to both target search and cleavage mechanisms.

## Evolution of the CRISPR-Cas systems

There are unifying themes in CRISPR-Cas evolution. These include contributions from MGEs, duplication of *cas* genes, and recombination of CRISPR-Cas modules to acquire variable functions (134, 323). The adaptation module is uniformly present in the diverse CRISPR-Cas systems, except in type IV systems. It encodes the conserved proteins Cas1 and Cas2, along with subtype-specific accessory proteins such as Cas4 or Csn2 (296, 324, 325). Cas1 proteins and the CRISPR repeat are thought to have evolved from self-synthesizing transposons called casposons (326).

The Class 1 systems have shown a predominant presence of RNA Recognition Motif (RRM)-containing proteins that is proposed to have divergently evolved to play diverse enzymatic roles or to serve in an RNA-binding capacity (15, 104, 143, 327). Within this system, the type III systems are believed to be the ancestor that evolved from abortive infection modules due to the production of cOA by Cas10 that triggers cell growth arrest by Csm6 (Fig. 1*B*) (15, 304). Some type III subtypes (A, B, and D) may have acquired the reverse transcriptase associated with the Group II intron to enable spacer acquisition from RNA (3, 104, 328, 329, 330). Type I systems are thought to have evolved from type III by the capture of Cas3 as an external component and replacement of Cas10 by Cas8 that lacks the ability to synthesize cOA (104), perhaps to create a module that will facilitate processive DNA degradation without any associated signaling events. Type IV systems are also thought to have evolved from type III due to similarities in protein components. Interestingly, type IV systems are predominantly found in plasmids, rather than in bacterial genomes (15, 104, 331). The proposed role of type IV systems is to clear heterologous plasmid infections in order to promote self-plasmid propagation.

In Class 2 systems, the signature proteins Cas9 (type II) and Cas12 (type V) show commonality due to the presence of a RuvC-like domain, though with minimal sequence similarities, and with insertions within the RuvC domain (HNH in Cas9 and Nuc domain in Cas12) (134, 304). Cas9 and Cas12 were proposed to have independently evolved from different protein subfamilies encoded by IS200/605-like transposons (134, 323). Cas9 is believed to have evolved from the IscB (Isc: Insertion Sequences Cas9-related) subfamily (104, 144, 332) and the diverse Cas12 subtypes are thought to have evolved from the TnpB (Transposon-associated RNA guided nuclease) subfamily (15, 304, 323). Structural and functional convergences are seen in Cas9 and Cas12a despite their diverged sequence composition (143, 327). Interestingly, Cas12k belonging to the CAST system has an inactive nuclease that is colocalized with the components necessary for transposition within the CRISPR-Cas locus. Cas12k-gRNA complex enables PAM-specific DNA targeting, which triggers the transposition of the cargo into the target DNA (Fig. 2*C*) (35, 37, 333, 334, 335). CAST systems were also identified in some subtypes of the type I system (I-B1, I-B2 and I-F3) (34, 336, 337, 338, 339). The loss of nuclease activity in Cas12k and Cas3, representing two different Classes, may point to instances of convergent evolution where the loss of CRISPR interference is being co-opted for transposition.

Cas13 of type VI systems and certain ancillary nucleases associated with type III systems (Csm6 and Csx1) possess the RNase domain HEPN and have similarities with the toxin module of the toxin-antitoxin (TA) systems (279, 323). The complexity of the HEPN domain of Cas13 compared to the simplicity of this domain in the TA systems has led to the proposal that Cas13 might have evolved from toxins related to the abortive infection system (24, 304, 340). The TA system has contributed to another aspect of the CRISPR system due to the evolution of Cas2 in the adaptation module from VapD toxin (104, 296).

Finally, bacteriophages and archaeal viruses have acquired CRISPR-Cas systems to promote their replication within the host (for example, through spacers that target phage inhibitory chromosomal island of the host to induce the lytic cycle) (341, 342, 343) or enabled protection from other viruses infecting the same host (40, 344, 345), emphasizing the dynamics of transferring and acquiring genomic content between bacteria and MGEs in shaping CRISPR-Cas diversity (304, 346).

## Summary and future perspectives

CRISPR-Cas systems are remarkable adaptive immune systems present in bacteria and archaea that have revolutionized biotechnology and genome editing applications, including gene therapy. This diverse system is not only essential for immune protection from invading MGEs, but also useful for controlling native gene expression (50, 303, 347, 348, 349, 350, 351, 352, 353). CRISPR-Cas systems possess several features that are related to eukaryotic immune systems, such as similarities based on cOA signaling and nucleotide-dependent immune responses in eukaryotes (354, 355, 356). RNA-guided endonucleases that are similar to CRISPR-Cas systems, called the Fanzor proteins, are also found in the eukaryotic transposon regions (357, 358). These similarities again point to the unity in the usage of RNA-guided mechanisms across all domains of life. Interestingly, the diverse CRISPR systems are adapted to provide a range of protection such as cleavage of the invading MGE, degradation of both DNA and RNA transcripts of the invader (type III systems), usage of the CRISPR system to only repress transcription (Cas12c system) (155), and finally programmed cell death/dormancy through activation of ancillary proteins (50, 181, 293). The use of RNP for immune protection is quintessential and can be attributed to the ability of RNA molecules to provide self-*versus* non-self-discrimination based on sequence complementarity and its ability to promote large-scale conformational changes (359).

From a molecular perspective, CRISPR-Cas systems are extraordinary in representing a complex organization ranging from multiple protein subunits (Class 1) to adapting single protein subunits (Class 2) to perform the shared function of RNA-guided nucleic acid-targeting and cleavage. CRISPR-Cas systems provide several examples of evolutionary convergence, for example, type III-E systems possessing features representative of both Class 1 and Class 2 systems. Within these unified mechanisms, we observe divergence, as exemplified in the type V RuvC active site that has been adapted to cleave different types of nucleic acids and the dimeric existence of HEPN domains in type VI systems (Table 1).

Even with existing rich information on CRISPR-Cas systems, there are several unknowns that need to be addressed to gain deeper insights into the diversity of CRISPR-Cas systems as new systems are being identified by metagenomic analyses. Assessing subtype diversity within the Class 1 systems in terms of helical filament organization, targeting mechanisms, and evolutionary correlations will be a profitable future direction for Class 1 systems. In addition to subunit composition differences among the different subtypes, several mechanistic details are lacking for each Class 1 type. Some open questions are the diversity of Cas3 in terms of its existence as single *versus* split genes; differences in type I interference mechanisms due to retaining *versus* recruiting Cas3 to Cascade; the diversity of type III subtype’s interference mechanisms ranging from DNA to RNA to protein cleavage; details of signaling mechanisms and resetting of secondary responses in type III; and the interference mechanisms of type IV systems and the role of their unique signature proteins toward interference. The simplicity of Class 2 systems with only one protein being involved in surveillance and interference complexes contributed to our superior understanding of Class 2-mediated targeting and cleavage mechanisms. Future studies should include more subtypes and more orthologs from each subtype to decipher differences in their molecular mechanisms. For example, the significance of the differences in domain lengths between type II subtypes; the application of currently available mechanism from *S. pyogenes* Cas9 to all Cas9s; the diversity of type V subtypes in terms of RNA requirements and differences in the type of nucleic acid being targeted by type V systems; structure and mechanisms of diverse type VI subtypes and differences in their PFS requirements; and secondary cellular effects following target RNA cleavage by type VI systems will be some avenues to pursue Class 2 systems. Unraveling these fundamental mechanisms and identifying diversity in terms of assembly, cleavage mechanisms, and fidelity, while establishing parallels with other RNA-guided enzymes, will drive improvements in the currently existing tools and the development of novel genome tools in the years to come.

Apart from the aforementioned future directions, there are other aspects that were not covered in this review that will also be at the forefront of CRISPR systems and CRISPR biology in the future. These will include understanding the physiological roles of CRISPR-Cas systems that deviate from the established models (*e.g.*, type IV and V); promotion of bacterial virulence by the CRISPR-Cas systems; regulation of CRISPR-Cas expression and the fitness-cost benefits of maintaining an active CRISPR-Cas system; the coexistence of several CRISPR-Cas types within a bacterial/archaeal genome; the decision by the cell to use targeted invader cleavage *versus* altruistic cell death; and the use of anti-CRISPR proteins and RNAs by phages to regulate CRISPR defense.

## Conflict of interest

The authors declare that they have no conflicts of interest with the contents of this article.
